# An Association Between Long-Term Exposure to Ambient Air Pollution and Mortality From Lung Cancer and Respiratory Diseases in Japan

**DOI:** 10.2188/jea.JE20100098

**Published:** 2011-03-05

**Authors:** Kota Katanoda, Tomotaka Sobue, Hiroshi Satoh, Kazuo Tajima, Takaichiro Suzuki, Haruo Nakatsuka, Toshiro Takezaki, Tomio Nakayama, Hiroshi Nitta, Kiyoshi Tanabe, Suketami Tominaga

**Affiliations:** 1 Cancer Information Services and Surveillance Division, Center for Cancer Control and Information Services, National Cancer Center, Tokyo, Japan; 2 Environmental Health Sciences, Tohoku University Graduate School of Medicine, Sendai, Japan; 3 Aichi Cancer Center Research Institute, Nagoya, Japan; 4 Department of Cancer Control and Statistics, Osaka Medical Center for Cancer and Cardiovascular Diseases, Osaka, Japan; 5 School of Nursing, Miyagi University, Miyagi, Japan; 6 Department of International Island and Community Medicine, Graduate School of Medical and Dental Sciences, Kagoshima University, Kagoshima, Japan; 7 Environmental Health Sciences Division, National Institute for Environmental Studies, Tsukuba, Japan; 8 Environmental Chemistry Division, National Institute for Environmental Studies, Tsukuba, Japan

**Keywords:** air pollution, lung neoplasms, nitrogen dioxide, particulate matter, sulfur dioxide

## Abstract

**Background:**

Evidence for a link between long-term exposure to air pollution and lung cancer is limited to Western populations. In this prospective cohort study, we examined this association in a Japanese population.

**Methods:**

The study comprised 63 520 participants living in 6 areas in 3 Japanese prefectures who were enrolled between 1983 and 1985. Exposure to particulate matter less than 2.5 µm in aerodynamic diameter (PM_2.5_), sulfur dioxide (SO_2_), and nitrogen dioxide (NO_2_) was assessed using data from monitoring stations located in or nearby each area. The Cox proportional hazards model was used to calculate the hazard ratios associated with the average concentrations of these air pollutants.

**Results:**

The 10-year average concentrations of PM_2.5_, SO_2_, and NO_2_ before recruitment (1974–1983) were 16.8 to 41.9 µg/m^3^, 2.4 to 19.0 ppb, and 1.2 to 33.7 ppb, respectively (inter-area range). During an average follow-up of 8.7 years, there were 6687 deaths, including 518 deaths from lung cancer. The hazard ratios for lung cancer mortality associated with a 10-unit increase in PM_2.5_ (µg/m^3^), SO_2_ (ppb), and NO_2_ (ppb) were 1.24 (95% confidence interval: 1.12–1.37), 1.26 (1.07–1.48), and 1.17 (1.10–1.26), respectively, after adjustment for tobacco smoking and other confounding factors. In addition, a significant increase in risk was observed for male smokers and female never smokers. Respiratory diseases, particularly pneumonia, were also significantly associated with all the air pollutants.

**Conclusions:**

Long-term exposure to air pollution is associated with lung cancer and respiratory diseases in Japan.

## INTRODUCTION

Many epidemiological studies of US and European populations have demonstrated an association between long-term exposure to ambient air pollution and mortality from lung cancer, cardiovascular diseases (CVDs), and respiratory diseases.^1^^–^^16^ However, these findings might not apply to Asia, where both the levels and constituents of air pollutants may differ from those in the West. For example, while the annual average ambient concentration of sulfur dioxide (SO_2_) in Tokyo during 2000–2005 was below 10 µg/m^3^ (3.8 ppb)—which was similar to levels in the least polluted cities of Europe, such as Copenhagen and Barcelona—the annual average ambient nitrogen dioxide (NO_2_) concentration in Tokyo was 60 µg/m^3^ (31.9 ppb), a value higher than those in the most polluted cities of Europe, such as Paris and Athens (unit conversion factors are based on the same citation).^17^ This raises the possibility that the magnitude of the mortality association in Asian populations differs from those in the United States and Europe.

National air quality monitoring networks have been established in Japan, and data on concentrations of SO_2_, NO_2_, particulate matter (PM), and other substances have been available in selected areas for several decades. In the present study, we examined the long-term effect of air pollution on mortality from lung cancer and respiratory diseases, using data obtained from a large-scale population-based Japanese cohort study of individuals enrolled between 1983 and 1985.

## METHODS

### Study population and baseline survey

We used data collected by the prospective Three-prefecture Cohort Study, which was conducted in 8 selected areas in Miyagi (Sendai city and Wakuya/Tajiri towns), Aichi (Nagoya city and Inuyama city), and Osaka (Osaka city, Nose, Kanan, and Kumatori towns) prefectures.^18^ The study areas in each prefecture were selected as a set of polluted and control areas. Specifically, the cities of Sendai, Nagoya, and Osaka were selected as the polluted areas, and the other areas were selected as the control areas. The study areas were chosen because they have national air pollution monitoring stations and well-managed cancer surveillance systems. The study population was defined as all residents in these areas aged 40 years or older. Participants were enrolled between 1983 and 1985 (February 1983 in Nose town, January-February 1984 in Sendai city and Wakuya/Tajiri towns, October-November 1984 in Osaka city, November-December 1984 in Kanan town, February-March 1985 in Kumatori town, July-August 1985 in Inuyama city, and October-November 1985 in Nagoya city). A self-administered questionnaire was distributed to 118 820 individuals identified based on residence registries in cooperation with the municipal government of each area, and responses were returned by 100 615 (84.7%). Individuals were excluded from the study if they had resided in the study areas for less than 10 years (*n* = 19 542) or provided incomplete answers to questions related to smoking status, pack-years (ever smokers only), smoking status of family members, frequency of vegetable and fruit consumption, or use of indoor charcoal or briquette braziers (*sumi* or *rentan* in Japanese) for heating (*n* = 17 553). The final analytic cohort comprised 63 520 participants (30 035 men and 33 485 women). Details of the study areas and participants are summarized in Table 1. The study was approved by the institutional review board of the National Cancer Center, Japan.

**Table 1.** Average air pollution levels and participant characteristics in the 6 study areas

			Miyagi prefecture		Aichi prefecture		Osaka prefecture	
					
			Wakuya/Tajiri towns (entire towns)	Sendai city (6 areas in Aoba and Miyagino wards)	Inuyama city (2 areas in the city)	Nagoya city (5 areas in Chikusa ward)	Nose/Kanan/ Kumatori towns (entire towns)	Osaka city (Higashinari ward)
Year of baseline survey			1984	1984	1985	1985	1983–1985^a^	1984
Number of participants in collected datasets			14 571	16 774	12 001	21 514	18 608	17 147
Number of participants in analytic cohorts			7813	9924	7917	13 653	10 490	13 723
Age at baseline; mean (SD)			56.8 (11.3)	57.5 (11.2)	56.2 (11.3)	57.7 (11.1)	55.9 (11.5)	57.6 (11.3)
40–49 years			30.4%	28.0%	34.0%	27.0%	35.5%	28.4%
50–59 years			33.1%	32.4%	31.1%	33.0%	30.9%	31.6%
60–69 years			20.3%	23.4%	20.3%	23.1%	18.6%	22.9%
≥70 years			16.2%	16.1%	14.6%	16.9%	15.1%	17.2%
Person-years of follow-up			71 579	80 927	70 819	114 497	94 917	117 599
% move-out during follow-up			2.9%	22.4%	8.0%	20.3%	5.5%	11.9%
**Number of deaths**								
Lung cancer			49	60	58	132	74	145
Respiratory diseases^b^			78	116	69	126	120	181
All causes			973	935	789	1333	1033	1624

**10-year average air pollution levels**								
SPM (µg/m^3^)	1974–1983		24.0	44.8	46.3	49.7	36.0	59.9
	1984–1993		21.9	29.0	37.4	43.7	36.2	45.0
PM_2.5_ (µg/m^3^)^c^	1974–1983		16.8	31.4	32.4	34.8	25.2	41.9
	1984–1993		15.3	20.3	26.2	30.6	25.3	31.5
SO_2_ (ppb)	1974–1983		2.4	12.0	9.5	10.4	13.5	19.0
	1984–1993		2.3	5.5	6.8	7.7	6.3	10.6
NO_2_ (ppb)	1974–1983		1.2	18.3	13.6	20.3	14.6	33.7
	1984–1993		2.6	16.1	16.0	23.9	16.0	33.0

**Results of baseline survey (1983–1985)**								
Smoking status (%)								
Current smokers^e^			30.3%	29.1%	35.6%	30.1%	35.1%	33.9%
Former smokers^e^			9.0%	14.4%	15.4%	17.4%	9.9%	13.9%
Amount of smoking (pack-years)								
Current smokers; mean (SD)^f^			31.5 (17.4)	32.0 (19.6)	33.8 (19.4)	34.7 (22.3)	34.8 (19.6)	34.5 (20.8)
Former smokers; mean (SD)^f^			27.9 (20.5)	29.4 (24.5)	29.4 (28.0)	30.3 (28.2)	28.5 (23.4)	29.8 (26.5)
Passive smoking (%)								
Currently from family members^e^			62.4%	46.2%	48.4%	39.7%	54.3%	50.4%
During childhood from parents^e^			71.4%	75.1%	74.3%	75.3%	79.3%	82.6%
Daily green and yellow vegetable consumption (%)^e^			52.1%	58.0%	41.8%	48.3%	36.6%	34.4%
Daily consumption of other vegetables (%)^e^			75.5%	67.7%	63.3%	62.1%	49.3%	41.9%
Daily fruit consumption (%)^e^			64.9%	65.0%	34.8%	54.4%	49.8%	47.2%
Use of indoor charcoal or briquette braziers for ​ heating (%)^e^			44.8%	7.1%	3.0%	1.5%	10.1%	6.3%
Occupation with potential risk (%)^d,e^			29.7%	24.7%	40.5%	27.1%	32.0%	36.3%
History of respiratory diseases (%)^e^			6.1%	6.9%	8.6%	10.4%	7.6%	6.6%
Health insurance type (%)								
National health insurance^e^			67.8%	44.2%	42.1%	42.2%	47.1%	56.2%
Government/union-managed health insurance^e^			21.1%	38.8%	48.8%	47.9%	41.9%	40.1%
Mutual aid associations health insurance^e^			9.0%	14.7%	8.2%	9.0%	10.0%	2.2%
Others			2.1%	2.3%	0.9%	0.9%	0.9%	1.5%

NO_2_, nitrogen dioxide; PM_2.5_, particulate matter <2.5 µm in aerodynamic diameter; SO_2_, sulfur dioxide; SPM, suspended particulate matter.

^a^1983 in Nose, 1984 in Kanan, and 1985 in Kumatori towns.

^b^For respiratory diseases, numbers of deaths were counted after excluding 4970 participants with a previous diagnosis of pneumonia, asthma, chronic bronchitis, emphysema, or pneumoconiosis.

^c^Estimated by multiplying the level of SPM by 0.7.

^d^Experience of occupation with potential risk of exposure to gases, fumes, or dust.

^e^Significant difference among areas (chi-square test; *P* < 0.0001).

^f^Significant difference among areas (ANOVA; *P* < 0.0001).

### Follow-up

The follow-up period was defined as 10 years from the baseline survey in each study area (through October 1995 at the latest). Vital status and out-migration were confirmed through registries kept by the local governments. Causes of death were confirmed by vital statistics obtained with official permission, and coded according to the International Classification of Diseases, 9th revision (ICD-9). Only the underlying cause of death was used. The endpoint was defined as lung cancer death during the observation period (ICD-9: 162). Deaths from respiratory diseases (ICD-9: 460–519) were also analyzed. We did not analyze other causes of death because we lacked baseline data for potential confounding factors.

### Air pollution data

**Figure 1.**
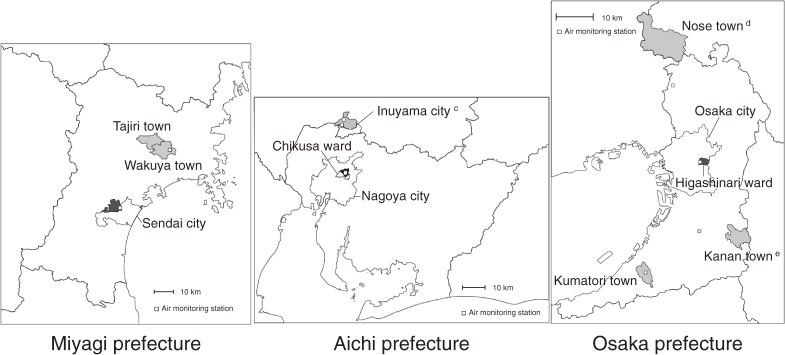
Geographical distribution of the study areas and air monitoring stations.^a,b^ ^a^Study areas are shaded in black (urban) or gray (rural). ^b^The name and boundaries of each area are those at the time of the baseline survey. ^c^There were 2 air monitoring stations in Inuyama city. ^d^The air monitoring station for Nose town is located to the south. ^e^The air monitoring station for Kanan town is located to the west.

Since the 1970s, there has been a network of ambient air monitoring stations in Japan operated by the Ministry of Environment (formerly the National Environment Agency) and local governments. The annual mean concentrations measured from 1974 to 1983 at an air monitoring station in or nearby each study area were used as surrogate indicators of individual exposure levels. Figure 1 shows the geographical distribution of the study areas and the air monitoring stations. All air monitoring stations were located within the study area, except those for Nose town and Kanan town in Osaka prefecture, which were located nearby. For Inuyama city, there were 2 monitoring stations, and the average values obtained from the 2 stations were used. The distance from the population center of each study area to the monitoring station was 6.5 km for Wakuya/Tajiri towns, 0.9 km for Sendai city, 1.7 km for Nagoya city, 1.4 km and 1.7 km for Inuyama city, 0.8 km for Osaka city, 15.7 km for Nose town, 10.7 km for Kanan town, and 0.4 km for Kumatori town.

The concentrations of SPM, SO_2_, and NO_2_ were measured by β-ray absorption, conductometry, and absorption spectrophotometry, respectively. In Japan, SPM is defined as particles with an aerodynamic diameter of ≤10 µm by the 100% cutoff point, which corresponds to approximately PM_7.0_ by the 50% cutoff point. In the 2 areas of Miyagi prefecture, SPM concentrations were measured in part by gravimetry incorporated into a low-volume sampler, rather than by β-ray absorption (1974–1986 in Wakuya/Tajiri towns and 1974–1985 in Sendai city). The SPM concentrations for these periods were estimated using the concentration ratios between the 2 measurement methods calculated for the subsequent years through 1996.

Many epidemiological studies have reported the health effects of ambient PM ≤2.5 µm in aerodynamic diameter (PM_2.5_),^4^^,^^9^^,^^11^^,^^13^^,^^15^^,^^16^ some of which suggested a stronger association with the fine fraction of PM than with the coarse fraction.^4^^,^^11^ Because we did not have long-term concentration data for PM_2.5_, we estimated PM_2.5_ concentrations by converting SPM concentrations using a single ratio, as in previous studies.^2^^,^^19^ We examined the PM_2.5_/SPM ratio in our study areas during a short period (2 weeks) in 1997; the ratio ranged from approximately 0.6 to 0.8. A similar range was observed in the annual average concentration data at the monitoring stations in the 3 prefectures from 2001 to 2005. The temporal correlation coefficient between the PM_2.5_ and SPM concentrations calculated from the data near Osaka city in 1974–1977 was greater than 0.90. Based on these data, we assumed that the PM_2.5_ concentration in each of the study areas was well correlated with the SPM concentration and that variations in the PM_2.5_/SPM ratio would be small across the study areas. Thus, we used the PM_2.5_ concentrations estimated by multiplying the SPM concentrations by a ratio of 0.7.

In the statistical analysis, the data from Nose, Kanan, and Kumatori towns were pooled and the average concentrations across those areas were used because they had similar air concentration levels (the average concentrations for the 1974–1993 period were: Nose 32.2 µg/m^3^, Kanan 37.7 µg/m^3^, and Kumatori 38.3 µg/m^3^ for SPM; Nose 7.8 ppb, Kanan 11.9 ppb, and Kumatori 10.0 ppb for SO_2_; Nose 15.4 ppb, Kanan 15.1 ppb, and Kumatori 15.5 ppb for NO_2_).

### Statistical analysis

Person-years of follow-up were calculated for all participants from the date of the baseline questionnaire to whichever of the following occurred first: the end of the 10-year follow-up, date of death, or date of moving out of the study area. Person-years for participants who died from causes other than those being analyzed were treated as being censored at the time of death. The Cox proportional hazards model was used to calculate the hazard ratio (HR) and 95% confidence interval (CI). The CI was calculated using the sandwich variance estimate to adjust for correlated observation within each study area.^20^

For the analysis of deaths from lung cancer, the HRs were adjusted for sex, age (continuous), smoking status (current, former, never), pack-years (<10, 10–19, ≥20), smoking status of family members living together (current smoking/no current smoking), daily green and yellow vegetable consumption (yes/no), daily fruit consumption (yes/no), and use of indoor charcoal or briquette braziers for heating (yes/no). Green and yellow vegetable consumption and fruit consumption were added to the adjusted variables because fruit and foods containing carotenoids have been judged as “probably protective” against lung cancer by the World Cancer Research Fund and the American Institute for Cancer Research.^21^ Use of indoor charcoal or briquette braziers for heating was adjusted for because household combustion of coal has been classified as carcinogenic to humans, especially with respect to lung cancer, by the International Agency for Research on Cancer.^22^ In our preliminary analysis, we confirmed that the results were unchanged after adjustment for use of oil or gas heaters without a flue instead of or in addition to use of charcoal or briquette braziers. Another model was applied to additionally adjust for the smoking status of the parents during the childhood of the participants (smoking/nonsmoking), daily consumption of vegetables other than green and yellow vegetables (yes/no), occupation (experience in occupations with potential exposure to gases, fumes, or dust or not), and health insurance as an indicator of socioeconomic status (4 categories: national health insurance, government or union-managed health insurance, mutual aid associations health insurance, others). All adjusted variables were based on the baseline survey. Analyses stratified by sex and smoking status and analyses excluding participants with a history of respiratory diseases were also performed. In the analyses stratified by sex and smoking status, we examined only male current smokers, male former smokers, and female never smokers, because the numbers of participants in the other strata were small.

Two approaches were used to evaluate the effects of air pollution.^4^ First, dummy variables for the study area were included, using the least polluted area, namely, Wakuya/Tajiri towns, as the reference category. Multivariate-adjusted HRs for each of the 6 areas were plotted against the average pollution levels in those areas. Second, area-specific concentrations of each pollutant were included directly in the models, and multivariate-adjusted HRs were estimated according to a 10-unit increase in air pollution levels. The average concentrations during the 10-year exposure time window (1974–1983) before the baseline survey were used for the analysis, and the subsequent 10-year average concentrations (1984–1993) corresponding to the follow-up period of our cohort were also analyzed. We only applied single-pollutant models because the number of measurement stations was small and the concentrations of different pollutants were spatially correlated (>0.80).

In the analysis of deaths from respiratory diseases, the HRs by 10-unit increase in air pollution levels were calculated after adjustment for sex, age (continuous), smoking status (current, former, never), pack-years (<10, 10–19, ≥20), smoking status of family members living together (current smoking/no current smoking), use of indoor charcoal or briquette braziers for heating (yes/no), and occupation (experience in occupations with potential exposure to gases, fumes, or dust or not). Participants with a previous diagnosis of pneumonia, asthma, chronic bronchitis, emphysema, or pneumoconiosis were excluded from the analysis (*n* = 4970). Analyses stratified by sex and smoking status were also performed. In all analyses, SAS version 8.02 (SAS Institute Japan Ltd.) was used to estimate HRs and CIs.

## RESULTS

### Air pollution data

The 10-year average concentrations (1974–1983) of SPM, PM_2.5_, SO_2_, and NO_2_ across the 6 study areas were 24.0 to 59.9 µg/m^3^, 16.8 to 41.9 µg/m^3^, 2.4 to 19.0 ppb, and 1.2 to 33.7 ppb, respectively (inter-area range). The average concentrations of these 4 air pollutants during the subsequent 10-year period (1984–1993) were 21.9 to 45.0 µg/m^3^, 15.3 to 31.5 µg/m^3^, 2.3 to 10.6 ppb, and 2.6 to 33.0 ppb, respectively (inter-area range). The area-specific air pollution levels during the 2 consecutive 10-year periods are shown in Table 1. The concentrations of all pollutants were highest in Osaka city and lowest in Wakuya/Tajiri towns for both 10-year periods. In all 6 study areas, the concentration of SO_2_ was lower by 0.1 to 8.4 ppb during the second 10-year period as compared with the first 10-year period. In all the study areas, excluding Nose/Kanan/Kumatori towns, the concentration of SPM was lower by 2.1 to 15.8 µg/m^3^ during the second period as compared with the first period. The concentrations of NO_2_ during the first and second periods were similar; the differences ranged from −2.2 to 3.6 ppb (second period minus first period).

**Figure 2.**
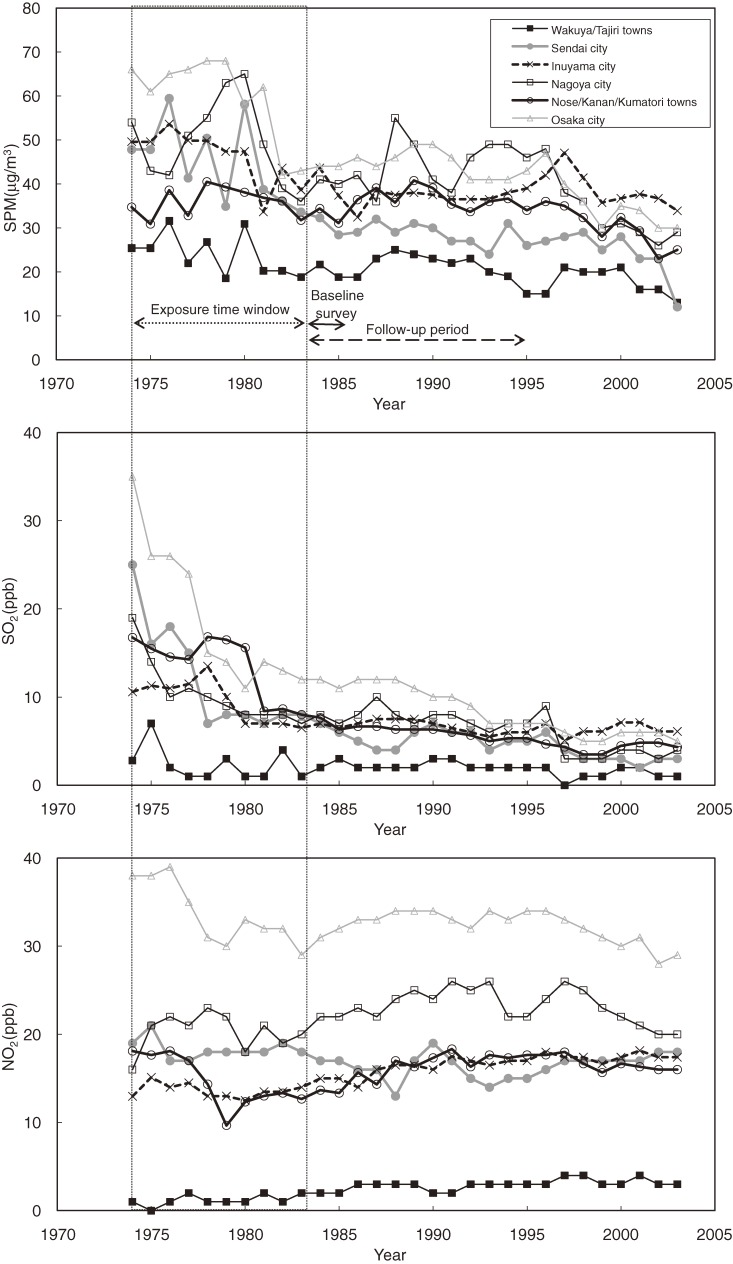
Annual trends in air pollutant levels from 1974 through 2003 in the 6 study areas. NO_2_, nitrogen dioxide; SO_2_, sulfur dioxide; SPM, suspended particulate matter.

Figure 2 shows the annual trends for the 3 air pollutants. The concentrations of SPM decreased during the early 1980s, especially in heavily polluted areas such as Sendai city, Nagoya city, and Osaka city. SO_2_ concentrations markedly decreased in the late 1970s, especially in Sendai city and Osaka city, and continued to decrease slowly thereafter. NO_2_ concentrations remained stable throughout the observation period. Significant temporal correlations were observed between the area-pooled concentrations of SO_2_ and NO_2_ within the exposure time window (1974–1983) (Pearson correlation coefficient = 0.76, *P* = 0.01). The temporal correlations between the concentrations of SPM and SO_2_ and between SPM and NO_2_ were weak (Pearson correlation coefficient: 0.47 [*P* = 0.17] and 0.26 [*P* = 0.48] respectively).

### Baseline characteristics

Area-specific baseline characteristics are shown in Table 1. The prevalence of current smokers ranged from 29% to 36%, and the prevalence of former smokers ranged from 9% to 17%. Pack-years were distributed within small ranges: 32 to 35 for current smokers and 28 to 30 for former smokers. Approximately half of the participants were exposed to passive smoking from current family members, and over 70% of the participants had been exposed to passive smoking from parents during childhood. The prevalence of the use of charcoal or briquette braziers for heating was over 40% in Wakuya/Tajiri towns, which are located in a cold rural region, and 10% or lower in the other study areas.

### Association between air pollution and lung cancer

**Figure 3.**
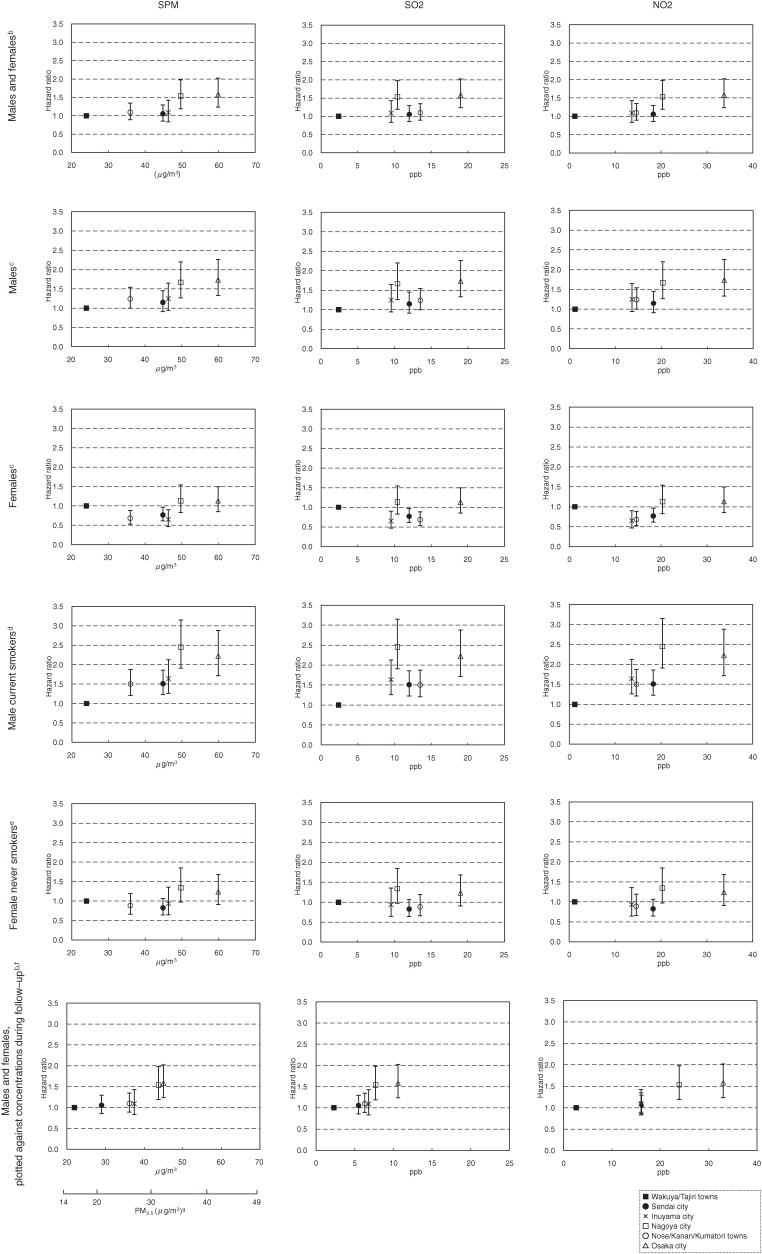
Adjusted hazard ratios for lung cancer mortality in the 6 study areas, plotted against average air pollutant concentrations.^a^ NO2, nitrogen dioxide; PM2.5, particulate matter <2.5 µm in aerodynamic diameter; SO2, sulfur dioxide; SPM, suspended particulate matter. ^a^10-year average concentrations (1974–1983) before the baseline survey are used as the horizontal axis, unless otherwise specified. Hazard ratios were calculated using Wakuya/Tajiri towns as a reference. Vertical bars indicate the 95% confidence intervals of the hazard ratios. ^b^Adjusted for sex, age (continuous), smoking status (current, former, or never), pack-years (0 to <10, 10 to <20, ≥20), smoking status of family members (current smoking/no current smoking), daily green and yellow vegetable consumption (yes/no), daily fruit consumption (yes/no), and indoor charcoal or briquette braziers used for heating (yes/no). ^c^Adjusted for age (continuous), smoking status (current, former, or never), pack-years (0 to <10, 10 to <20, 20 to <30, 30 to <40, ≥40 for men; 0 to <10, 10 to <20, ≥20 for women), smoking status of family members (current smoking/no current smoking), daily green and yellow vegetable consumption (yes/no), daily fruit consumption (yes/no), and indoor charcoal or briquette braziers used for heating (yes/no). ^d^Adjusted for age (continuous), pack-years (0 to <10, 10 to <20, 20 to <30, 30 to <40, ≥40), smoking status of family members (current smoking/no current smoking), daily green and yellow vegetable consumption (yes/no), daily fruit consumption (yes/no), and indoor charcoal or briquette braziers used for heating (yes/no). ^e^Adjusted for age (continuous), smoking status of family members (current smoking/no current smoking), daily green and yellow vegetable consumption (yes/no), daily fruit consumption (yes/no), and indoor charcoal or briquette braziers used for heating (yes/no). ^f^10-year average concentration (1984–93) during the follow-up period was used as the horizontal axis. ^g^Estimated by multiplying the level of SPM by 0.7.

The number of cause-specific deaths and person-years of follow-up for the 6 study areas are shown in Table 1. In total, 6687 deaths were observed, including 518 deaths from lung cancer, during an average follow-up of 8.7 years. Figure 3 shows the adjusted HRs of lung cancer mortality relative to the least polluted area, plotted against the 10-year average concentration levels of the air pollutants (1974–1983). For men and women combined, a significant increase in risk was observed in Nagoya city and Osaka city at a level of 50 µg/m^3^ or higher for SPM (equivalent to 35 µg/m^3^ or higher for PM_2.5_) and at a level of 20 ppb or higher for NO_2_. The HR point estimates for these 2 areas were approximately 1.5.

When stratified by sex, the results for men were similar to those for the entire population. Among women, although HRs were not significantly higher even in the most polluted area, there was a gentle concentration-response gradient regardless of whether the analysis was or was not limited to never smokers. For male current smokers, a significant increase in risk was observed even in the second least polluted area at an SPM level of approximately 36 µg/m^3^ (equivalent to 25 µg/m^3^ for PM_2.5_), an SO_2_ concentration of 10 ppb, and an NO_2_ concentration of 14 ppb, with HR point estimates of approximately 1.5. The highest HR point estimate for male current smokers was close to 2.5.

The bottom row of Figure 3 shows the results obtained when the exposure time window was shifted to the 10-year average concentration levels during the follow-up period (1984–1993). For SPM and SO_2_, the distribution of 6 areas moved towards the lower half of the concentration ranges, and, accordingly, a significant increase in risk was observed for an SPM concentration of approximately 45 µg/m^3^ (equivalent to 30 µg/m^3^ for PM_2.5_) and an SO_2_ concentration of 7.5 ppb. By contrast, for NO_2_, the concentration ranges in the 6 areas were similar to those in 1974–1983, and a significant increase in risk was observed at a concentration of approximately 25 ppb.

Table 2 shows the adjusted HR for lung cancer associated with a 10-unit difference in the 10-year average air pollution levels (1974–1983). A significant association with lung cancer mortality was observed for all 4 pollutants after adjustment for sex, age, smoking status, pack-years, smoking status of family members, daily green and yellow vegetable consumption, daily fruit consumption, and use of indoor charcoal or briquette braziers for heating (base model). The point estimates of the increased risk associated with a 10-unit increase in the concentrations were 16%, 24%, 26%, and 17% for SPM (µg/m^3^), PM_2.5_ (µg/m^3^), SO_2_ (ppb), and NO_2_ (ppb), respectively. These increases in risk remained even after additional adjustment for the smoking status of parents during the participants’ childhood, daily consumption of vegetables other than green and yellow vegetables, occupation, and health insurance (full model), although the association was not significant for SO_2_. The significant associations with the 4 air pollutants were observed even after exclusion of participants with a history of respiratory diseases.

**Table 2.** Adjusted HRs for lung cancer associated with a 10-unit increase in the average concentration of air pollutants^a^

Model description	Category	Number of deaths	Person-years	SPM (µg/m^3^)		PM_2.5_ (µg/m^3^)^b^		SO_2_ (ppb)		NO_2_ (ppb)	
			
				HR	95% CI	HR	95% CI	HR	95% CI	HR	95% CI
Sex- and age-adjusted	(All)	518	550 339	1.18	(1.13–1.24)	1.27	(1.19–1.36)	1.36	(1.20–1.54)	1.20	(1.17–1.24)
Base model^c^	(All)	518	550 339	1.16	(1.08–1.25)	1.24	(1.12–1.37)	1.26	(1.07–1.48)	1.17	(1.10–1.26)
Full model^d^	(All)	421	472 762	1.16	(1.06–1.25)	1.23	(1.09–1.38)	1.19	(0.97–1.45)	1.15	(1.06–1.24)
By sex^e^	Male	407	257 120	1.17	(1.09–1.26)	1.26	(1.14–1.39)	1.30	(1.12–1.52)	1.18	(1.11–1.26)
	Female	111	293 219	1.12	(0.99–1.26)	1.17	(0.98–1.39)	1.11	(0.88–1.40)	1.13	(1.01–1.27)
By sex and smoking status^f^	Male current smokers	292	146 031	1.23	(1.14–1.34)	1.35	(1.20–1.52)	1.36	(1.06–1.75)	1.23	(1.12–1.35)
	Male former smokers	90	63 092	1.08	(0.83–1.39)	1.11	(0.77–1.60)	1.25	(0.75–2.10)	1.13	(0.88–1.44)
	Female never smokers	73	254 145	1.11	(1.01–1.22)	1.16	(1.02–1.33)	1.09	(0.92–1.29)	1.11	(1.02–1.20)
Base model, excluding participants with a history of respiratory diseases^c,g^	(No history of respiratory disease)	438	509 369	1.15	(1.06–1.26)	1.23	(1.08–1.39)	1.22	(1.02–1.45)	1.16	(1.07–1.25)
Base model, using average concentrations during the follow-up period^c,h^	(All)	518	550 339	1.27	(1.14–1.41)	1.41	(1.21–1.64)	1.97	(1.41–2.75)	1.21	(1.10–1.33)

CI, confidence interval; HR, hazard ratio; NO_2_, nitrogen dioxide; PM_2.5_, particulate matter <2.5 µm in aerodynamic diameter; SO_2_, sulfur dioxide; SPM: suspended particulate matter.

^a^10-year average concentrations (1974–1983) before baseline survey were used, unless otherwise specified.

^b^Estimated by multiplying the level of SPM by 0.7.

^c^Adjusted for sex, age (continuous), smoking status (current, former, or never), pack-years (0 to <10, 10 to <20, ≥20), smoking status of family members (current smoking/no current smoking), daily green and yellow vegetable consumption (yes/no), daily fruit consumption (yes/no), and indoor charcoal or briquette braziers used for heating (yes/no).

^d^Base model, with additional adjustment for smoking status of parents in childhood (current smoking/no current smoking), daily consumption of vegetables other than green and yellow vegetables (yes/no), occupation (experience in occupation with potential exposure to gases, fumes, or dust or not), health insurance (4 categories). 9289 participants were excluded because of missing data for smoking status of parents, non-green/yellow vegetable consumption, occupation, and/or health insurance.

^e^Base model, excluding adjustment for sex. Pack-years were adjusted with different categories for males and females (0 to <10, 10 to <20, 20 to <30, 30 to <40, ≥40 for males, 0 to <10, 10 to <20, ≥20 for females).

^f^Base model, excluding adjustment for sex and smoking status. Pack-years were adjusted using 5 categories for male current or former smokers (0 to <10, 10 to <20, 20 to <30, 30 to <40, ≥40).

^g^4970 participants with a previous diagnosis of pneumonia, asthma, chronic bronchitis, emphysema, or pneumoconiosis were excluded.

^h^Base model, using the 10-year average concentrations (1984–1993) during the follow-up period.

Men showed a significant increase in the risk for all 4 air pollutants, with point estimates ranging from 17% to 30%. For women, the corresponding increases in risk ranged from 11% to 17%, although the increase was significant only for NO_2_. However, analyses limited to female never smokers revealed significant excess risk associated with SPM, PM_2.5_, and NO_2_. The increase in risk among male current smokers was significantly higher, with point estimates ranging from 23% to 36%, while no significant increase in risk was observed for male former smokers. The interactions between smoking status and air pollutant concentrations were not statistically significant in men or women (*P* > 0.07 for men and *P* > 0.26 for women).

When we incorporated the average air pollution levels during the follow-up period in the model (1984–1993), instead of those for the period before baseline (1974–1983), the excess risk associated with air pollution increased, with point estimates ranging from 21% to 97%.

### Association between air pollution and respiratory diseases

Table 3 shows the adjusted HRs for mortality from respiratory diseases associated with 10-unit differences in the air pollution levels. A significant association was observed between mortality from respiratory diseases and the concentrations of all 4 pollutants. This association was observed in both sexes, and in male current smokers and female never smokers. The association was similar when the exposure time window was shifted to the 10-year average concentration levels during the follow-up period (1984–1993). In analysis stratified by disease, the association was significant for pneumonia, but not for chronic obstructive pulmonary disease (COPD).

**Table 3.** Adjusted HRs for respiratory diseases associated with a 10-unit increase in the average concentration of air pollutants^a^

Model description	Category	Number of deaths	Person-years	SPM (µg/m^3^)		PM_2.5_ (µg/m^3^)^b^		SO_2_ (ppb)		NO_2_ (ppb)	
			
				HR	95% CI	HR	95% CI	HR	95% CI	HR	95% CI
Sex- and age-adjusted	(All)	677	509 369	1.08	(0.98–1.18)	1.11	(0.98–1.27)	1.38	(1.23–1.55)	1.13	(1.08–1.19)
Multivariate model^c^	(All)	677	509 369	1.11	(1.03–1.20)	1.16	(1.04–1.30)	1.43	(1.33–1.54)	1.16	(1.12–1.21)
By sex^e^	Male	417	236 047	1.07	(1.00–1.15)	1.11	(1.00–1.22)	1.30	(1.16–1.47)	1.11	(1.05–1.18)
	Female	260	273 322	1.19	(1.07–1.32)	1.28	(1.10–1.49)	1.68	(1.40–2.01)	1.25	(1.18–1.33)
By sex and ​smoking status^f^	Male current smokers	200	134 750	1.15	(1.03–1.28)	1.22	(1.05–1.42)	1.52	(1.35–1.71)	1.21	(1.11–1.31)
	Male former smokers	130	56 849	0.89	(0.79–1.01)	0.85	(0.71–1.01)	0.88	(0.66–1.18)	0.90	(0.80–1.01)
	Female never smokers	218	238 431	1.19	(1.09–1.30)	1.29	(1.14–1.46)	1.63	(1.44–1.84)	1.25	(1.20–1.30)
Pneumonia^c^	(All)	512	509 369	1.12	(1.03–1.21)	1.17	(1.04–1.32)	1.45	(1.34–1.57)	1.16	(1.12–1.21)
COPD^c^	(All)	64	509 369	0.92	(0.78–1.08)	0.89	(0.70–1.12)	1.32	(0.88–1.98)	1.03	(0.93–1.15)
Respiratory ​diseases, using ​average concentrations ​during the follow-up period^c,g^	(All)	677	509 369	1.08	(0.89–1.31)	1.12	(0.85–1.46)	1.62	(1.22–2.15)	1.14	(1.06–1.23)

CI, confidence interval; HR, hazard ratio; NO_2_, nitrogen dioxide; PM_2.5_, particulate matter <2.5 µm in aerodynamic diameter; SO_2_, sulfur dioxide; SPM, suspended particulate matter.

^a^10-year average concentrations (1974–1983) before the baseline survey were used, unless otherwise specified. 4970 participants with a previous diagnosis of pneumonia, asthma, chronic bronchitis, emphysema, or pneumoconiosis were excluded.

^b^Estimated by multiplying the level of SPM by 0.7.

^c^Adjusted for sex, age (continuous), smoking status (current, former, or never), pack-years (0 to <10, 10 to <20, ≥20), smoking status of family members (current smoking/no current smoking), indoor charcoal or briquette braziers used for heating (yes/no), and occupation (experience of occupation with potential exposure to gases, fumes, or dust or not).

^e^Multivariate model, excluding adjustment for sex. Pack-years were adjusted with different categories for males and females (0 to <10, 10 to <20, 20 to <30, 30 to <40, 40 for males, 0 to <10, 10 to <20, ≥20 for females).

^f^Multivariate model, excluding adjustment for sex and smoking status. Pack-years was classified into 5 categories for male current and former smokers (0 to <10, 10 to <20, 20 to <30, 30 to <40, ≥40).

^g^Multivariate model, using the 10-year average concentrations (1984–1993) during the follow-up period.

## DISCUSSION

This large-scale prospective cohort study demonstrated associations between long-term exposure to both particulate and gaseous ambient air pollution and an elevated risk of lung cancer mortality in the Japanese population, after controlling for potential confounding factors. The observed increase in risk ranged from approximately 16% to 26% for a 10-unit increase in air pollution levels. These values were generally comparable to those reported in previous studies conducted in the United States and European countries. For example, in previous studies, point estimates of the adjusted relative risks for a 10-µg/m^3^ increase in PM_2.5_ concentration ranged from 1.06 to 1.39 (calculated from published values, when necessary).^2^^,^^9^^,^^11^^,^^13^^,^^15^ Our results suggest that the concentration-response gradient observed in US and European populations can also be applied to Japan, despite the differences in air pollution levels and constituents^17^ and disease and risk distributions.^23^^,^^24^

Men, especially current smokers, had higher HRs for lung cancer associated with air pollution in the present study, even after controlling for smoking status and pack-years. Although the interactions between smoking status and air pollutant concentrations were not statistically significant, the stronger association for current smokers raises the question of whether the observed association between lung cancer and air pollution was confounded by unmeasured exposure to smoking. However, our results remained unchanged when we used a model that additionally controlled for age at initiation of smoking (data not shown), and a significant increase in risk was also observed for female never smokers. Although it is possible that active smoking and long-term exposure to air pollution have an additive or synergistic effect on lung cancer, previous studies are not consistent regarding the different effects of air pollution on lung cancer with respect to sex and smoking status. European studies showed weaker associations between air pollution and lung cancer for males^13^ and current and former smokers.^2^ These results cannot be interpreted in a straightforward manner because they were obtained without controlling for smoking status^13^ or amount of smoking.^2^ The American Cancer Society (ACS) study, which was controlled for active and passive smoking levels, reported stronger associations between air pollution and lung cancer in males, whereas the associations were more evident in former smokers.^15^

As compared with the effects of smoking, the excess risk of lung cancer associated with air pollution was small. In the present study, the multivariate-adjusted HR for lung cancer mortality in current smokers relative to never smokers was 4.8 (data not shown), which is consistent with the results of previous large-scale and meta-analytic studies conducted in Japan.^24^^,^^25^ The maximum difference in PM_2.5_ concentration during the 30-year observation period was 40 µg/m^3^ (approximately 50 µg/m^3^ in Osaka city versus 10 µg/m^3^ in Wakuya/Tajiri towns). Similarly, the maximum differences in SO_2_ and NO_2_ concentrations were 35 ppb and 40 ppb, respectively. These differences in concentrations, combined with the observed relative risks for a 10-unit increase, correspond to a 1.9- to 2.4-fold increase in the risk of lung cancer.

With regard to respiratory mortality, previous findings regarding the effect of long-term exposure to ambient air pollution have been somewhat inconsistent. The Harvard Six Cities Study, the Adventist Health Study of Smog (AHSMOG), and several European studies reported positive associations for PM_2.5_,^1^^,^^2^^,^^9^^,^^11^^,^^13^ but only some of these associations were significant.^13^ For SO_2_, many studies showed a relative risk close to unity.^1^^,^^2^^,^^14^ For NO_2_, both significant^2^^,^^13^ and null^1^ associations have been reported. Some of those reports should be interpreted with caution, however. The AHSMOG was limited to nonsmoking males and females belonging to a specific religious group,^1^^,^^11^ and 2 European studies did not adjust for smoking status^13^ or amount of smoking.^2^ In the present study, we controlled for potential confounding factors for respiratory diseases and observed significant increases in risk due to particulate and gaseous air pollution.

Respiratory diseases represent a variety of medical conditions that have different etiologies. The present study observed a significant effect of air pollution on pneumonia, but not COPD. However, data on disease-specific mortality were limited. The majority of the observed deaths from respiratory diseases were from pneumonia (76%); COPD accounted for only 9% of deaths. Although we only extracted respiratory diseases as the underlying cause of death, this category might have included respiratory complications of other underlying diseases or might have failed to identify underlying chronic respiratory diseases. Many of the studies cited above used the same broad definition of respiratory diseases as that of the present study (ie, ICD-9: 460–519).^1^^,^^2^^,^^11^^,^^14^ Indeed, the AHSMOG adopted an even broader outcome, namely, “any mention of respiratory mortality,” which included the contributing cause of death as well as the underlying cause of death.^1^^,^^11^ In contrast, a Norwegian study focused on COPD mortality and observed a significant association with PM and NO_2_.^13^ Similar disease-specific studies need to be accumulated to address the question of whether long-term exposure to air pollution affects chronic respiratory mortality.

The major strength of the present study is its prospective cohort design, which allowed for direct control of individual risk factors, particularly smoking status and level of exposure to smoking. The participants of the present study were enrolled on a population basis, and the response rate was high. Furthermore, our long-term exposure data allowed us to use the pre-baseline time window of ambient air pollution data.

There are several sources of uncertainty in the present study. First, we used mean ambient concentrations in or nearby each study area, instead of individual exposure levels. As was the case in many studies using the same exposure assessment, there could be spatial variability within an area, as well as differences among individuals in time spent outdoors.^26^

Second, it is difficult to determine which time period was most important in relation to health effects, because there were no substantial changes in relative air pollution levels in the study areas over the observation period. This difficulty is also true of previous studies; the ranking of cities from low to high pollution levels changed little across different time windows in the ACS study^15^ and the Harvard Six Cities Study.^27^ As illustrated in Figure 2, air pollution levels sharply differed before and after the baseline survey. When we shifted the exposure time window from the 10-year period before the baseline survey (1974–1983) to the subsequent 10-year period (1984–1993), the HRs for lung cancer mortality increased, especially in relation to SPM, PM_2.5_, and SO_2_ levels. This is because the absolute levels of these air pollutants decreased over time, while the relative levels for these 2 periods remained almost constant (the values for the spatial correlation between the 2 time periods for each pollutant were >0.85). The difficulty in distinguishing between the effects of past and recent exposure is also relevant to identification of the latency period. The observed increase in risk associated with air pollution levels during the 10 years before the baseline survey suggests that the effects occurred after a certain lag period. However, we cannot exclude the possibility of the effect occurring within a shorter lag period of 1 or 2 years.^28^

A third source of uncertainty is that we estimated PM_2.5_ concentrations by converting SPM concentrations using a single ratio (0.7), as in previous studies.^2^^,^^19^ As mentioned above, in our partial monitoring data, the PM_2.5_/SPM ratio ranged from approximately 0.6 to 0.8. If we assume that between-area variations in the ratio were within this range, there would be no substantial differences in the ordering of the study areas, because the concentrations of SPM were distributed over a wide range. At selected European sites, high correlations were found between coarse and fine PM concentrations (PM_2.5_ and PM_10_; *R*^2^ > 0.98),^29^ and PM_2.5_/PM_10_ ratios were uniform.^30^ However, a slight tendency towards higher ratios at rural background sites as compared with urban traffic sites was also reported.^30^ In the United States, PM_2.5_/PM_10_ ratios in the eastern part of the country were double those in the Southwest, and a poor correlation was observed between PM_2.5_ and PM_10_ concentrations in the West (eg, *r* < 0.5 in the upper Midwest).^31^ The components of PM may also vary by time.^31^ In our study areas, the concentration of substances such as SO_2_ decreased during and after the exposure window (Figure 2), which could have affected the components of PM and the coarse and fine fractions of PM. Because we had limited data on the concentrations of different particulate sizes in our study areas, it remains uncertain how well the SPM concentration represented the variability of the PM_2.5_ concentration. As mentioned in the Methods above, SPM concentration was measured differently in 2 study areas of Miyagi prefecture. Although this might be another source of uncertainty, excluding those 2 areas did not change our results substantially: the HR for a 10-unit increase in SPM concentration was 1.17 (95% CI, 1.12–1.24) for lung cancer and 1.09 (0.92–1.29) for respiratory diseases.

Fourth, pollutant concentrations were correlated with each other. The spatial correlation between different air pollutants within the exposure time window was very high (>0.80). In addition, SO_2_ and NO_2_ are potential constituents of ambient PM, and their concentration levels vary over time. Therefore, it is very difficult to isolate the health effects of individual pollutants. To understand the biologic mechanisms underlying the increased risk of a specific disease, or to specify potential determinant substances, additional toxicological research is needed.

Finally, our results could have been affected by the exclusion of participants with missing data. We confirmed that the unadjusted HRs were similar when these data were included and excluded from the analysis. Smoking status was unknown among approximately two thirds of the participants whose data were excluded. However, we performed a sensitivity analysis that defined the participants with unknown smoking status as either current or never smokers and confirmed that the effect of the exclusion was negligible. Although there were participants who had moved out of the study areas during the follow-up period, this was considered to have occurred in an unselective way because the characteristics of disease-specific mortality in our analytic cohort were consistent with the reported vital statistics of the study areas.^32^

In conclusion, our large-scale prospective cohort study revealed an association between long-term exposure to ambient air pollution and mortality from lung cancer and respiratory diseases in Japan.
